# Video Playback Speed Influence on Learning Effect From the Perspective of Personalized Adaptive Learning: A Study Based on Cognitive Load Theory

**DOI:** 10.3389/fpsyg.2022.839982

**Published:** 2022-05-12

**Authors:** Chuan-Yu Mo, Chengliang Wang, Jian Dai, Peiqi Jin

**Affiliations:** ^1^School of Education and Music, Sanming University, Sanming, China; ^2^College of Educational Science and Technology, Zhejiang University of Technology, Hangzhou, China; ^3^College of Foreign Languages, Zhejiang University of Technology, Hangzhou, China

**Keywords:** personalized learning, video playback speed, learning effect, cognitive load, online learning

## Abstract

Following the COVID-19 pandemic, online learning has become a new mode of learning that students must adapt to. However, the mechanisms by which students receive and grasp knowledge in the online learning mode remain unknown. Cognitive load theory (CLT) offers instructions to students considering the knowledge of human cognition. Therefore, this study considers the CLT to explore the internal mechanism of learning under the online mode in an experimental study. We recruited 76 undergraduates and randomly assigned them to four groups in which they will watch videos at four different kinds of speed (1.0× or 1.25× or 1.5× or 2× speed). The study observed and analyzed how video playback speed affected students' learning and cognitive load to obtain the following results: (1) Video playback speed significantly influenced the students' learning effect. The best effect was observed at the speed of 1.25× and 1.5×. (2) The speed that affected the learning effect best differed according to the students' learning abilities. High-level group students performed best at the speed of 1.5×, whereas low-level group students performed best at the speed of 1.25×. (3) The 1.5× speed showed significant differences in the learning effect by students' majors. This indicates that the cognitive load of liberal arts students increased greatly at this speed. (4) A change in playback speed has a significant impact on the cognitive load. Accelerated playback speed increases the cognitive load of students. The highest learning effect is observed under medium cognitive load.

## Introduction

Currently, the continuous emergence of online educational platforms and the abundant high-quality online learning resources have rendered diversified and up-to-date online instructional videos an indispensable part of the education system (Cigdem, 2014; Bates, 2015). Moreover, the post-pandemic period events led to the rapid promotion of online teaching and increased the students' online learning time (Ferri et al., 2020). Thus, students are required to adapt to the new online learning methods and environment with the support of new technology. In recent years, some scholars have explored the difference in cognitive load between online and traditional learning methods. They found that online learning brought more cognitive load to students than offline learning (Bradford, 2011; Skulmowski and Xu, 2021). Previous studies have also examined the influence of playback speed on the learning effect (Pastore, 2010; Ritzhaupt et al., 2015). However, contrary to the findings of previous studies, the learning modes have been found to negatively change during the COVID-19 pandemic period (Salta et al., 2021). However, students are forced to adopt such unfamiliar method of learning online, which takes a lot of time and energy for them to get used to (Jin et al., 2021; Lin et al., 2021). Therefore, we need to explore the changes in cognitive load when students use videos to study through experimental research conducted during the pandemic.

Studies in the literature have examined the acceptance, satisfaction, and availability of online education (Dhawan, 2020; Han and Sa, 2021; Mo et al., 2021). However, another serious problem remains to be considered: how to better utilize the online educational platforms once both the teachers and students accept the online learning system. Compared with the offline mode, online learning platforms provide students with more freedom to control their learning paces by allowing them to choose suitable video playback speeds (Singh and Thurman, 2019). This personalized adaptive learning (PAL) mode of online courses ensures the learners' freedom and independence and provides them with learning assistance consistent with their characteristics (Peng et al., 2019). However, online learning environments may also present problems of information overload and mismatch of resources while providing massive learning content (Raj and Renumol, 2018; Sweller et al., 2019). Therefore, students can adjust the properties of online resources and thus improve their learning efficiency, control their cognitive load, and increase the learning effect (Katrin et al., 2020). Moreover, during the post-pandemic period, students could conform to the online education mode and obtain the ideal learning effect. In sum, we mainly answer the following questions in this paper:

(1) What speed should students choose for their online courses?
(2) Does the learning effect differ by the playback speeds? Does the variable of learning ability influence the learning effect?
(3) Does the learners' cognitive load differ by the playback speed?
(4) Is there a correlation between students' learning effect and cognitive load?

## Literature Review

### Current Studies of Personalized Adaptive Learning

Personalized learning specifically refers to the learning method by which the learning speed and teaching methods can be optimized, considering the needs of each learner. Students may have different learning goals and prefer different education methods and learning order (King and South, 2017). Adaptive learning adjusts the instructions based on the learning data obtained from students (Becker et al., 2017). The recent development in technology not only improved the adaptability of personalized learning but also personalized it, promoting the emergence of personalized adaptive learning (PAL) (Peng et al., 2019).

Following a decade of research on adaptive learning, Xie et al. (2019) conclude that PAL is a research topic centered on students' learning. Using a literature survey method, Bernacki et al. (2021) summarize the theoretical PAL guidance methods used to evaluate the learning practice. From the results of past studies based on the analysis of traditional learners' characteristics (such as learning styles and prerequisite knowledge levels) in adaptive learning (Normadhi et al., 2019), this study refers to the research experiment of Ritzhaupt et al. (2015) on video playback speed. We choose majors, learning ability, cognitive load, and other individual student characteristics as the main feature information and data sources of PAL. The purpose of this study was to assist students in using adaptive learning at a personalized video playback speed.

### Cognitive Load Theory

Cognitive load theory (CLT) was first proposed by Sweller (1988); it then developed rapidly during the 1990s. Cognitive load is an important concept relating to the total amount of information the human information-processing system can deal with. It mainly consists of loads of information stored and processed in working memory (Sweller, 2008). CLT assumes two types of cognitive load: intrinsic cognitive load and extraneous cognitive load. The intrinsic cognitive load is determined by the level of learner expertise and complexity of the learning content, while the extraneous cognitive load is determined by the teaching methods and organization and property of instructional material (Kalyuga, 2011; Kalyuga and Sweller, 2014). CLT mainly explores the relationship between extraneous cognitive load and learning effect.

Many scholars have applied CLT to educational research. For example, Lai et al. (2019) applied CLT to develop an AR-based science learning system. They later found through experiments that this system significantly decreased students' perceptions of extraneous cognitive load. Chen et al. (2017b) proposed instructional design principles based on CLT to structure online learning platforms such as massive open online courses (MOOCs). Eitel et al. (2020) examined the relationship between self-control, cognitive load, and self-regulated learning, to find that self-management is linked with self-regulated learning and cognitive load. CLT has also been employed to study instructional videos. Carrying out experiments based on CLT, Altinpulluk et al. (2020) found that dividing an instructional video into several meaningful parts could reduce learners' cognitive load. However, studies have rarely focused on the effect of video playback speed on cognitive load. Therefore, considering the past studies, we further verify, expand, and enrich the connotations of CLT through experiments.

### Research on Video Playback Speed

Following the development of online education, instructional videos have become an indispensable part of students' learning. Moreover, 2012 is known as the first year of MOOCs (Pappano, 2012). Therefore, scholars are increasingly examining online teaching and instructional videos (Christensen et al., 2013; Hone and El Said, 2016; Littenberg-Tobias and Reich, 2020). There are many fields in the research of instructional videos. For example, some scholars focus on the interaction between learners' behaviors and videos to understand why learners skip, reflect, and pause when watching videos (Brinton et al., 2015). Other scholars examine the video properties such as subtitles, bullet-screen comments, length of time, and playback speed (Evans et al., 2016; Tarchi et al., 2021).

Video playback speed is an important property of instructional videos which has attracted the attention of many scholars. Ritzhaupt et al. (2015) examined the influence of three speeds (1.0×, 1.25×, and 1.5×) on the learning effect of participants. They found that the difference in video playback speed had little influence on the learning effect, although it significantly affected the satisfaction level and cognitive load of participants, who found less satisfaction with 1.5× speed than with 1.0× speed. Furthermore, through experiments, Pastore (2010) found that an increase in speed decreased the learning effect. He thus proposed a hypothesis: Learners fail to conduct schema construction well when the learning time is shortened because of the accelerated speed, and this lessens the effectiveness of learning compared to that at normal speed.

Some studies focused on the impact on learners when the video playback speed is lowered. Experiments showed that learners were more satisfied with normal speed than 0.75× speed (Davis et al., 2021). Other studies found learners more likely to watch the entire videos when the video time is reduced by appropriately speeding up (Lang et al., 2020).

From previous studies, online learning platforms (such as MOOCs), found on a large scale, have collected huge amounts of learners' behavioral data, but have not innovated in-course design or restructured platform architecture based on the data, and have failed to solve some difficult problems of online education (Ross et al., 2014). Scholars have pointed out that although some online courses have been adjusted based on research, the video completion rate of courses on online platforms is still far below that of traditional courses (Gütl et al., 2014; Evans et al., 2016).

However, previous research on video properties such as playback speed does not integrate with PAL or explore how to employ the findings to PAL. Therefore, we first consider the utilization of personalized curricula as well as students' personalized learning characteristics and then study the influence of playback speed on learning effect and cognitive load.

## Methodology

### Participants

The study participants were 76 undergraduates from University A in Zhejiang Province. Following previous surveys, we screened out the students who studied MATLAB and selected those with some knowledge of computer programming (specifically, those who learned programming language or C + + or Python, following the specialized training plan of a university) but no idea of MATLAB. The demographics of the selected participants are given in Table 1.

**Table 1:** Basic information of participants.

	**Gender**		**Grade**				**Major**		
	**Male**	**Female**	**Freshman**	**Sophomore**	**Junior**	**Senior**	**Science**	**Engineering**	**Liberal arts**
*N*	33	43	24	39	10	3	38	20	18

### Instruments

Our experiment consisted of six parts.

#### Online Learning Questionnaire

This questionnaire collects the students' basic information such as gender, grade, major, usual playback speed, and most common learning platform. The questionnaire is also designed to investigate the students' knowledge about MATLAB. The students' characteristic information collected from the questionnaire provided the data required for the following experiments.

#### Prior Learning Ability Pre-test Scale

This part is designed considering the self-regulated learning ability scale proposed by Zhao et al. (2014) and hence has good content validity. The scale has 3 first-grade indices, 8 second-grade indices, and 22 score points; the full score is 100. Cronbach's alpha coefficient is used to examine the reliability of the scale. The alpha coefficient of the whole scale is found to be 0.830, indicating high reliability. The Kaiser–Meyer–Olkin (KMO) coefficient of the whole table is 0.818, indicating good construct validity of the scale.

#### Pre-test and Post-test Papers

This questionnaire has two parts consisting of 10 multiple-choice questions (5 points per question) and 5 blank-filling questions (10 points per question); the full score is 100 points. After designing the questionnaire, we conducted a difficulty test and time evaluation to ensure moderate difficulty and adequate time for the tests.

#### Teaching Videos

Prior online learning studies have pointed out that concise teaching videos are more helpful to students and shorter courses result in a higher completion rate (Evans et al., 2016). However, the standard video length has not been defined in prior studies. Scholars in a study on MOOCs found the average length of a MOOCs video to be about 691s (Da Silva et al., 2016). For the choice of teaching video in our experiment, we considered the following factors. We aimed to present a better experimental result and find the change in cognitive load under different speeds in detail, but as the video duration will be shortened as the speed increases, we chose a teaching video that was slightly longer than the average duration of the general online teaching videos. The video we used for the experiment is String Processing from the video series Scientific Computing and MATLAB language played on MOOCs in China University and BiliBili platforms. This video has 11 knowledge points and lasts for 16 min under normal speed. The teaching video is authoritative. It was made by four excellent teachers from Central South University and has been certified as a high-quality online opening course for the Chinese MOOCs platform. Since January 2018, the courses have served the public for several years, indicating their popularity. Teachers in this video speak at the normal speed of 210 words per minute on average. This is almost equal to the average speed of 212 words per minute in MOOCs (The video link is https://www.icourse163.org/course/CSU-1002475002?tid=1465171453).

#### Cognitive Load Scale

The cognitive load scale is based on the seven-point scale proposed by Hwang et al. (2013) and the research on the reliability and validity of cognitive load by Anmarkrud et al. (2019). The scale was then adjusted considering the reality and hence its validity. The scale has eight questions in total, each with seven answers ranging from “strongly disagree” = 1 to “strongly agree” = 7. The first five questions on mental load are designed to evaluate the effect of difficulty of knowledge on students' cognitive load. The last three questions, with mental effort as an index, evaluate the cognitive load effect in different ways of presentation and interpretation. Cronbach's alpha coefficient is used to examine the reliability of the scale. The alpha coefficient of the whole scale is found to be 0.942, indicating high reliability. The KMO coefficient of the whole table is 0.861, indicating good construct validity of the scale.

#### In-person Interviews

These interviews supplement the opaque data that could not be quantified in the above experiments. Qualitative interviews provide the personalized opinions of respondents as well as in-depth information on specific topics (Turner, 2010; Phillippi and Lauderdale, 2018). In line with the general principles of interviews in sociology (Bryman, 2016; Mann, 2016), 10 questions on cognitive load and satisfaction with videos are prepared for the interviewees.

### Experiment Design and Data Collection

This study chooses a random between-subjects design for the experiment (Lundstedt et al., 1998), following Ritzhaupt et al. (2015), and then adjusts it in accordance with the reality. The experiment comprises four parts: pre-experiment preparation, pre-test, formal experiment, and post-test and interviews. The experiment is carried out to study how the playback speed of online teaching videos affects students with different learning abilities. It also explores whether speeding up the teaching videos would lead to learners' cognitive overload, as well as factors such as learners' gender, grade, and prerequisite knowledge level that could affect the learning scores of students while watching online teaching videos.

Researchers investigated the common online platforms that college students use to learn and the usual speed they choose when watching teaching videos. This study tries to offer guidance to students on how to choose appropriate video speeds and provide online teaching platforms with related data, as well as provide guidance to teachers who make teaching videos.

#### Pre-experiment Preparation

We begin with screening out the students who do not meet the experimental requirements. Generally, participants are not allowed to pause and go back during the experiment for two reasons. First, in most schools adopting lifecasting classes, it is not easy for students to pause or return. Second, to ensure conviction of the experiment results, the learning course of the participants should be standardized with the same speed. Furthermore, to ensure success of the experiment and conviction of results, suspension and going back should be prohibited. Then, researchers explain the experimental process to the eligible participants. Lastly, participants answer the online teaching video learning questionnaire and provide their basic information such as major, grade, and common platform. The online learning questionnaire shows that no students use 0.75× speed to watch the teaching videos. Therefore, students are randomly divided into four groups by speed: normal speed (17 people), 1.25× speed (21 people), 1.5× speed (21 people), and 2× speed (17 people).

#### Pre-test

Before the experiment, participants are required to fill out the pre-test table to evaluate their learning ability. From the results, the participants are divided into high-level and low-level groups. The dividing line between the two groups is at 70 points (Zhao et al., 2014). After completing the pre-test table, participants are required to answer the pre-test questionnaire to test their knowledge about experimental videos; this would indicate the students' prerequisite knowledge level. The researchers then put the test paper away.

#### Formal Experiment

Participants are randomly divided into four groups by the four playback speeds. To make the online learning as real as possible, the researchers offer mobile phones, laptops, and headphones to the participants for use according to their learning habits. They are also given manuscript papers.

#### Post-test and Interviews

After they watch the teaching video, the participants are asked to answer the post-test paper immediately and complete it within 8 min (The time limit is based on an earlier survey of difficulty test and time evaluation). Cognitive load scales are then issued to participants for them to learn their cognitive load during the experiment. Later, 20 participants are randomly selected for interviews after the post-test. As one of the most natural and commonly accepted methods to collect information (Dörnyei, 2007), interviews can help researchers study the influence of playback speed on students' learning effect and cognitive load and provide specific information that could support the quantitative research.

The final step is to sort out and analyze the obtained data.

## Result and Discussion

The following data are collected for the experiment: participants' prerequisite knowledge level measured by pre-test scores, learning effect measured by post-test scores, and cognitive load measured by cognitive load scale scores. The experiment results were analyzed for the playback speed influence on learning effect and cognitive load with SPSS (Statistical Package for Social Sciences) 26.0 software.

### Playback Speed Influence on Learning Effect and Discussion

#### Influence of Different Speeds on Learning Effect

First, the participants' post-test scores are statistically analyzed by gender using the *t*-test, to ensure that the playback speed showed no significant difference in effect on learning performance by gender. Then, we use covariance analysis to process the data. The analysis results are presented in Table 2. The results show that prerequisite knowledge level is an irrelevant variable and that playback speed has a significant influence on learning performance (*F* = 4.505, *P* = 0.006). Furthermore, participants learn best at 1.25× speed (*M* = 80, *SD* = 12.649) and worst at 2.0× speed (*M* = 66.47, *SD* = 12.595). A comparative analysis shows no significant difference between 1.0× and other speeds in learning performance, no obvious difference between 1.25× and 1.5× speeds in learning performance, much better performance at 1.25× speed than at 2.0× speed (*p* = 0.003 <0.01), and much better performance at 1.5× speed than at 2.0× speed (*p* = 0.026 <0.05).

**Table 2:** Influence of video speed on learning effect shown by covariance analysis.

**Video Speed**	***N***	**Pre-test**	**Prerequisite Knowledge**	**SD**	***F***	***p***
1	17	27.64	72.35	11.472	4.505	0.006
1.25	21	19.05	80.00	12.649		
1.5	21	23.81	76.43	15.980		
2	17	15.59	66.47	12.595		

The main reason for the poor learning effect at 1.0× speed may be that students are distracted more easily and can experience learning burnout when teachers speak at a low speed. Moreover, students who easily acquire knowledge when teachers speak at a low speed show reduced self-efficiency of learning and a lack of learning motivation and enthusiasm. This presumption conforms to the conclusion of scholars that “slowing down the video speed reduces learners' satisfaction” based on empirical research (Gütl et al., 2014). According to the Yerkes–Dodson law, the learning effect would be low under a lower level of motivation in easier tasks (Broadhurst, 1959; Sari, 2020).

Previous studies have pointed out that students are more easily distracted when facing the screen than when facing teachers, implying that the learning motivation of students is significantly lower when learning online than when learning offline (Nashir and Laili, 2021). The interview shows that participants fail to increase their learning motivation at lower speed. Therefore, the negative impact of 1.0× speed on the learning effect is significant in online learning. The main factor for learners' poor performance at higher speed is the cognitive overload caused by fast speed. According to the CLT, when the playback speed is altered, the unit-time information flow transmitted to students through teaching videos changes as well. Furthermore, large amounts of information flowing into learners' minds in a short time will overload their information-processing channels. Unable to reasonably digest and absorb the relative knowledge, students would then have insufficient time to encode information into a complete and orderly schema construction (Sweller, 2008, 2011). Thus, the students' learning scores would decrease at 2.0× speed.

#### Influence of Different Speeds on Learning Effect and Major Differences

The post-test score *t*-test analysis results of the participants of different majors are given in Table 3. From the table, the various majors have no significant difference in the post-test scores at 1.0×, 1.25×, and 2.0× speeds. However, the majors show significant influence on the scores at 1.5× speed (*F* = 0.632, *P* = 0.027). Science and engineering participants (*M* = 80.63) show much higher scores than liberal arts participants (*M* = 63.00).

**Table 3:** Influence of video speed and majors on learning effect shown by *t*-test analysis.

**Video speed**	**Majors**	***N***	**Post-test**	***t***	***df***	***p***
1	Liberal arts	5	70.00	−0.534	4.525	0.722
	Science and engineering	12	73.33			
1.25	Liberal arts	2	65.00	−1.870	19	0.077
	Science and engineering	19	81.58			
1.5	Liberal arts	5	63.00	−2.394	19	0.027
	Science and engineering	16	80.63			
2	Liberal arts	6	68.33	0.439	15	0.667
	Science and engineering	11	65.45			

Along with the interviews, the data show no significant cognitive load of participants at 1.0× and 1.25× speeds due to slow video playback speed, whether they major in arts or science. All have a relatively good knowledge, so there is no significant difference in post-test scores. However, at 1.5× speed, accelerating the speed would increase the cognitive load of liberal arts participants and thus decrease the learning effect. Meanwhile, the cognitive load of science and engineering participants will be at a lower level, with their learning effect remaining higher. According to the expertise reversal and element interactivity effects (Sweller, 2020), the release of large amounts of professional expressions and knowledge in a short time will reduce the interaction of elements and result in the expertise reversal effect (Kalyuga et al., 2003; Chen et al., 2017a). Therefore, participants from different majors perform differently at 1.5× speed. The video playback speed at 2.0× is so fast that the cognitive load of participants majoring in science and engineering increases significantly. Thus, the post-test scores show little difference between the participants majoring in liberal arts and sciences.

#### Influence of Accelerated Speed on Learning Effect and Learning Ability Difference

**Figure 1:**
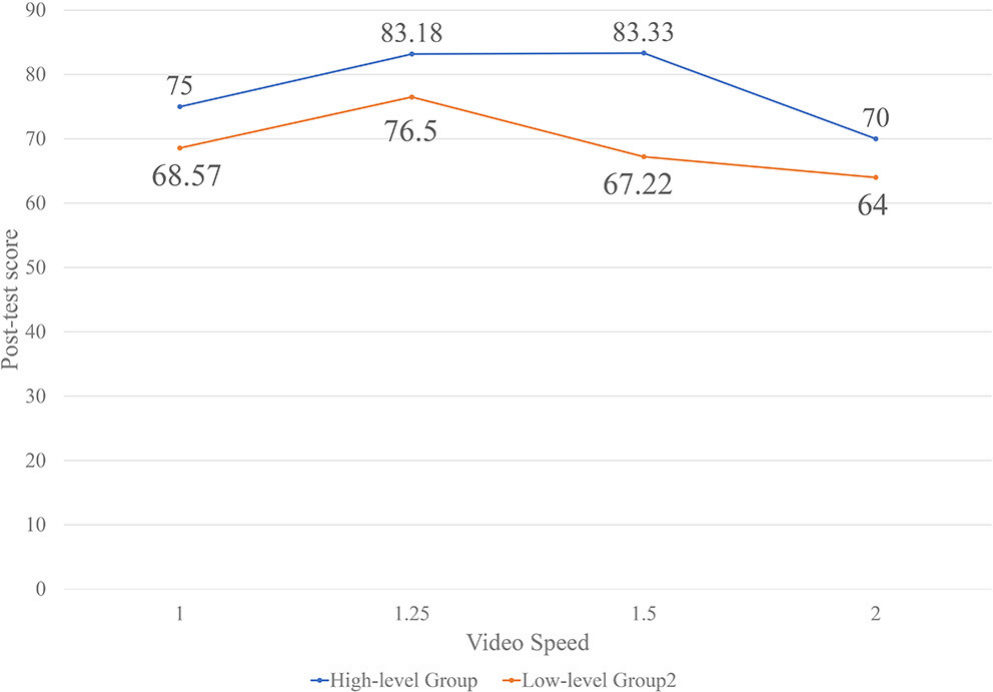
Post-test mean scores of different learning abilities.

From the participants' learning abilities reflected in the learning ability scale scores, the participants are divided into high-level and low-level groups. We explore the relationship between learning ability and the playback speed influence on the learning effect through a further data analysis using ANOVA with the two groups. The results are given in Table 4. The average post-test scores of different learning ability groups are visualized and displayed in Figure 1.

**Table 4:** Influence of video speed and learning ability on learning effect shown by covariance analysis.

**Groups**	**Video Speed**	***N***	**Post-test**	**SD**	**F**	***P***
High-level group	1.0	10	75.00	7.817	2.676	0.062
	1.25	11	83.18	11.017		
	1.5	12	83.33	13.707		
	2.0	7	70.00	14.720		
Low-level group	1.0	7	68.57	15.197	3.827	0.019
	1.25	10	76.50	13.945		
	1.5	9	67.22	4.640		
	2.0	10	64.00	10.541		

Both Table 4 and Figure 1 show no significant influence of playback speed on the learning effect in the high-level group. However, the low-level group's learning effect differs significantly between the different speeds. Thus, playback speed has little impact on the learning effect for students with higher learning ability, but greatly influences students with lower learning ability. The post-test scores of both the high-level and low-level groups are displayed in Figure 1. From the figure, when the speed changes from 1.0× to 2.0×, the low-level group shows greater volatility, indicating that their learning effect is significantly affected by the change in speed. Furthermore, the high-level participants perform best at 1.5× speed (*M* = 83.33), while the low-level subjects show the highest scores at 1.25× speed (*M* = 76.50).

From the data, we can infer that students with strong learning abilities adapt better to the different video speeds and show relatively stable scores. Furthermore, from the transient information effect and working memory depletion effect of CLT (Sweller, 2020), students with strong learning abilities can process longer instantaneous information and have larger short-term memory capacity. Therefore, when the video speed changes from 1.25× to 1.5×, the two groups show little difference in the learning effect. However, at 2.0× speed, the learning effect decreases significantly due to the upper limit of short-term memory capacity. Therefore, from the perspective of PAL, students should be aware of their learning ability when studying online and choose the video playback speed appropriate for them.

### Results and Discussion on Cognitive Load Effect

#### Influence of Accelerated Playback Speed on Cognitive Load

The cognitive load scale scores and accelerated speed are analyzed using the SPSS software; the results are given in Table 5. From the results, playback speed has a significant influence on cognitive load (*F* = 8.296, *P* = 0.000). With the increase in video playback speed, the cognitive load of participants increases greatly. Along with the above analysis of “the influence of playback speed on learning effect,” this shows that when the playback speed is altered, the information presented through video per unit-time changes as well. Therefore, the amount of information presented per unit-time directly affects students' cognitive load (Derry, 2020).

**Table 5:** Influence of video speed on cognitive load shown by covariance analysis.

**Video Speed**	***N***	**Cognitive load**	**SD**	***F***	***P***
1	17	38.71	6.273	8.296	0.000
1.25	21	43.48	6.047		
1.5	21	44.95	4.769		
2	17	47.94	4.943		

The teaching materials need to be set up and their related attributes adjusted to increase the amount of information presented per unit time. With the increase in video playback speed, the amount of information would also increase, which in turn would influence learners' external cognitive load (Sweller, 1988; Ritzhaupt et al., 2015), and finally, the acceleration would influence the learning effect.

#### Influence of Accelerated Playback Speed on Cognitive Load and Its Relation With Learning Ability

The data of playback speed and cognitive load with students' different learning abilities are analyzed using ANOVA; the results are displayed in Table 6. From the results, video playback speed has a significant influence on students' cognitive load for both the high-level and low-level groups. However, the cognitive load of the high-level group is lower than that of the low-level group at all speeds. Through further exploration, we find that the cognitive load scores (s = 44.42) of the high-level group are very close to those of the low-level group (s = 44.60) when both groups obtain the best learning effect (at 1.25× speed for the high-level group and 1.5× speed for the low-level group).

**Table 6:** Influence of video speed and learning ability on cognitive load shown by covariance analysis.

**Groups**	**Video speed**	***N***	**Mean cognitive load**	**SD**	***F***	***p***
High-level group	1.0	10	37.90	7.445	3.321	0.030
	1.25	11	42.45	5.803		
	1.5	12	44.42	5.600		
	2.0	7	46.71	5.619		
Low-level group	1.0	7	39.86	4.375	4.631	0.008
	1.25	10	44.60	6.415		
	1.5	9	45.67	3.571		
	2.0	10	48.80	4.517		

Along with the above analysis in section influence of accelerated playback speed on cognitive load and its relation with learning ability, this shows that the students' schematic understanding reaches the highest level under a medium-level cognitive load. Then, the students will not forget what they know because of the long explanation time and the cognitive load will not hinder them from processing the received information flow in time (Sweller et al., 2019; Curum and Khedo, 2021). Therefore, students would often have stronger learning motivation (Schiefele, 1991; Riswanto and Aryani, 2017), ensuring the learning effect. From the perspective of PAL, learners should find their cognitive load range from the cognitive load scale before taking up online learning; this is an important basis for students to select their appropriate video playback speed.

### Interviews and Discussion: Students' Views on Cognitive Load

To obtain more convincing experimental conclusions, this study adopted a combination of quantitative and qualitative methods (Schulze, 2003; Holland and Campbell, 2005). The researchers finally randomly interviewed 20 participants (each group has five participants) having different speeds on their cognitive load and satisfaction. From the results, the participants with lower cognitive load expressed higher satisfaction at 1.25× and 1.5× speeds, but experienced decreased satisfaction when watching the videos at 2.0× and 1.0× speeds.

During the interview, participant A, who watched the video at 1.0× speed, said that the teacher spoke relatively slowly at a normal speed and that he could be easily distracted without much learning motivation at this speed. Simple learning tasks arouse learning motivation with much difficulty and allow students to be easily distracted. Therefore, students do not learn efficiently at the normal speed. This finding strongly supports the experimental conclusion based on experimental data.

Participant B, who watched the video at 2.0× speed, said that the teacher spoke too fast; often, the next knowledge point came before he could grasp the last point. This was a tiring speed. His views reflect those of the participants who watched the video at 2.0× speed; these students bore a heavy cognitive load, failed to keep up with the teacher's speed, and thus had reduced learning satisfaction. This finding is consistent with our experimental data results showing poor learning effect at 2.0× speed.

The interview results and experimental data of this study strongly support the conclusions that “poor learning effect at lower speed is a result of simple learning tasks, which fail to stimulate learners' interest,” and that “an increase in speed also increases the cognitive load.” Therefore, students are supposed to take the initiative and find their own learning effect before taking up online learning and then make reasonable adjustments to their learning rhythm and playback speed.

## Conclusions, Implication, and Prospect

### Conclusion

Following the COVID-19 pandemic, the Ministry of Education of China asked schools at all levels to change the form of online teaching and learning classes. Therefore, unlike previous online learning, where students took the initiative to have classes online, this new method due to the pandemic introduced a passive learning mode. Against this backdrop, this study mainly discusses the influence of video playback speed on learning effect and cognitive load. From the study based on quantitative experiments and supplemented by qualitative interviews, we found that playback speed has a significant influence on the learning effect. Students show a much better learning effect at 1.25× and 1.5× speeds than at the other speeds; the effect is the lowest at 2× speed.

Compared with earlier studies, we focus on learning ability, which is a refinement of personalized property and explores the depth of this field. Previous studies often neglected the factor of students' learning ability, although this is not an irrelevant variable. Learning ability is of great importance closely related to the humanistic educational belief that “teachers should respect the personalized differences of students”. Therefore, we consider the learning ability of students as a variable in this study. This study has shown that different learning ability groups perform slightly differently. The high-level group learns best at 1.5× speed, whereas the low-level group learns best at 1.25× speed. Along with the interviews, this indicates that students with strong learning ability who can transfer information into schema adapt to accelerated speed better by altering their learning strategies. Furthermore, students of different majors perform differently at 1.5× speed. Science and engineering students show a significantly better learning effect than liberal arts students.

By studying the influence of playback speed on cognitive load, we could conclude that “cognitive load increases with the acceleration of playback speed.” Furthermore, we found that the high-level group and low-level group show almost similar cognitive load scores when they obtain their best learning effects. That is, learners achieve the best learning effect under appropriate cognitive pressure. This conclusion is consistent with the findings of studies on “the influence of playback speed on learning effect” (Queirós et al., 2017; Gläser-Zikuda et al., 2020), rendering the study results more convincing.

### Practical Implications

The purpose of studying the influence of video playback speed on students' learning effect and cognitive load is to promote students' PAL under the online learning environment. This study proposes suggestions and strategies to promote PAL from the following aspects.

First, course designers need to conduct pre-tests for students before they start learning online, to understand their learning habits and individual characteristics. Designers can recommend appropriate video playback speed for students from the obtained information. Furthermore, online course designers need to provide information on the teachers' average speaking speed to assist students to choose a suitable playback speed (Christ et al., 2017).

Second, considering the relationship between video playback speed and learning effect, learners need to choose appropriate speeds based on their individual situations, the difficulty of knowledge, and the attributes of the video itself. If learners cannot find the speed suitable for them, they are advised to begin at 1.25× speed and then adjust it according to the actual learning situation.

Third, considering the influence of speed on the learning effect of students with different learning abilities, learners should know their abilities from different aspects using the learning ability scale. Students should know their own strengths and weaknesses to enable them to choose their personalized video playback speed.

Furthermore, considering the relationship between playback speed and cognitive load, learners are advised to notice whether they have born too much cognitive load while seeking a high learning effect. Students should themselves take the initiative to understand their cognitive load using the cognitive load scale before learning online (Sweller, 1994; Mayer and Moreno, 2003; Schnotz and Kürschner, 2007). This will help them to adjust the playback speed and cognitive load appropriately. They should try to avoid decreases in their satisfaction levels due to fast playback speed, which could result in learning weariness and failure to keep learning.

### Limitations and Future Research

This work has several limitations that need to be addressed. We hope to supplement the present experimental conclusions in a future study.

First, we used only MATLAB language teaching videos as the experimental material and included only undergraduate students in the experiment. This lacked subject and crowd universality. However, this study provided guidance to students on the speed setting of various computer language online courses of universities and assisted them in PAL. To improve the universality of this study, future works should widen the range of participants and conduct experiments separately by type of course.

Second, our experimental scale limited the number of participants. Therefore, future studies should expand the experimental scale and thus increase the accuracy and reliability of the conclusions.

Furthermore, our experiment did not consider the influence of subtitles, video time, and other factors on the learning effect. This too remains to be explored in a future study. Moreover, following the COVID-19 pandemic, the learning period of most students has become significantly longer than earlier. Thus, learning time may be a major factor influencing learning effect and cognitive load. Future studies need to also explore whether the learning period affects the cognitive load.

With the development of PAL and individuation of learning resources, future studies need to explore the personalization of learning material and thus support the reforms in education informatization.

## Data Availability Statement

The original contributions presented in the study are included in the article/supplementary material, further inquiries can be directed to the corresponding authors.

## Author Contributions

The authors contributed equally to the conception of the idea, implementing and analyzing the experimental results, and writing the manuscript. All authors have read and agreed to the published version of the manuscript.

## Funding

This research was supported by the Starting Research Fund from the Sanming University (19YG15S) and Sanming University's Cultivate Fund for the National Social Science Foundation of China (PYS2108).

## Conflict of Interest

The authors declare that the research was conducted in the absence of any commercial or financial relationships that could be construed as a potential conflict of interest.

## Publisher's Note

All claims expressed in this article are solely those of the authors and do not necessarily represent those of their affiliated organizations, or those of the publisher, the editors and the reviewers. Any product that may be evaluated in this article, or claim that may be made by its manufacturer, is not guaranteed or endorsed by the publisher.
