# Corrigendum: Video playback speed influence on learning effect from the perspective of personalized adaptive learning: A study based on cognitive load theory

**DOI:** 10.3389/fpsyg.2022.947209

**Published:** 2022-11-04

**Authors:** Chuan-Yu Mo, Chengliang Wang, Jian Dai, Peiqi Jin

**Affiliations:** ^1^School of Education and Music, Sanming University, Sanming, China; ^2^College of Educational Science and Technology, Zhejiang University of Technology, Hangzhou, China; ^3^College of Foreign Languages, Zhejiang University of Technology, Hangzhou, China

**Keywords:** personalized learning, video playback speed, learning effect, cognitive load, online learning

In the original article, the original Table 3 was incorrectly included in the published article. Table 3 has now been removed from the article. The subsequent tables have been renumbered accordingly.

In addition a correction has been made to Result and Discussion, Results and Discussion on Cognitive Load Effect, Influence of Accelerated Playback Speed on Cognitive Load and Its Relation With Learning Ability, Paragraph 1. Instead of “Tables 6, 7,” it should be “Table 6.” The corrected paragraph is shown below:

The data of playback speed and cognitive load with students' different learning abilities are analyzed using ANOVA; the results are displayed in Table 6. From the results, video playback speed has a significant influence on students' cognitive load for both the high-level and low-level groups. However, the cognitive load of the high-level group is lower than that of the low-level group at all speeds. Through further exploration, we find that the cognitive load scores (s = 44.42) of the high-level group are very close to those of the low-level group (s = 44.60) when both groups obtain the best learning effect (at 1.25× speed for the high-level group and 1.5× speed for the low-level group).

The authors apologize for these errors and state that they do not change the scientific conclusions of the article in any way. The original article has been updated.

## Publisher's note

All claims expressed in this article are solely those of the authors and do not necessarily represent those of their affiliated organizations, or those of the publisher, the editors and the reviewers. Any product that may be evaluated in this article, or claim that may be made by its manufacturer, is not guaranteed or endorsed by the publisher.

